# Implication of the F-Box Protein FBXL21 in Circadian Pacemaker Function in Mammals

**DOI:** 10.1371/journal.pone.0003530

**Published:** 2008-10-27

**Authors:** Hugues Dardente, Jorge Mendoza, Jean-Michel Fustin, Etienne Challet, David G. Hazlerigg

**Affiliations:** 1 School of Biological Sciences, Aberdeen University, Aberdeen, Scotland, United Kingdom; 2 Institut de Neurosciences Cellulaires et Intégratives, Département de Neurobiologie des Rythmes, UMR7168, Strasbourg, France; National Institute on Aging, United States of America

## Abstract

In mammals, the circadian clock relies on interlocked feedback loops involving clock genes and their protein products. Post-translational modifications control intracellular trafficking, functionality and degradation of clock proteins and are keys to the functioning of the clock as recently exemplified for the F-Box protein Fbxl3. The SCF^Fbxl3^ complex directs degradation of CRY1/2 proteins and Fbxl3 murine mutants have a slower clock. To assess whether the role of Fbxl3 is phylogenetically conserved, we investigated its function in the sheep, a diurnal ungulate. Our data show that Fbxl3 function is conserved and further reveal that its closest homologue, the F-Box protein Fbxl21, also binds to CRY1 which impairs its repressive action towards the transcriptional activators CLOCK/BMAL1. However, while Fbxl3 appears to be ubiquitously expressed, Fbxl21 expression is tissue-specific. Furthermore, and in sharp contrast with Fbxl3, Fbxl21 is highly expressed within the suprachiasmatic nuclei, site of the master clock, where it displays marked circadian oscillations apparently driven by members of the PAR-bZIP family. Finally, for both Fbxl3 and Fbxl21 we identified and functionally characterized novel splice-variants, which might reduce CRY1 proteasomal degradation dependent on cell context. Altogether, these data establish Fbxl21 as a novel circadian clock-controlled gene that plays a specific role within the mammalian circadian pacemaker.

## Introduction

Endogenous circadian clocks generate rhythms of physiology and behaviour, and are synchronised to the environment through rhythmical stimuli, principally the light/dark cycle. These clocks allow organisms to anticipate and adapt to cyclical changes in environmental favourability.

In mammals, the circadian clock relies on interlocked feedback loops in which a heterodimer of transcription factors, CLOCK/BMAL1 (CLK/BM1), drives transcription of *Period1-3* (*Per1-3*) and *Cryptochrome1-2* (*Cry1-2*) genes through E-Box cis-elements. The PER1-3 and CRY1-2 proteins then act as transcriptional inhibitors of CLK/BM1. Apart from *Per1-3* and *Cry1-2*, the CLK/BM1 heterodimer acts on other genes, some of which are themselves clock components, such as the orphan nuclear receptors *Rev-erb α* or *Ror α*, while others are outputs from the clock, resulting in the regulation of cell- and tissue-specific processes [1]–[3]. The orphan nuclear receptors of the ROR and REV-ERB families act on DNA motifs known as ROREs to control rhythmic transcription of the *Bmal1* gene, with activating and repressive action, respectively [4]–[7]. Other ancillary interlocked feedback loops, such as that through which REV-ERBs and RORs impinge on the transcriptional control of *Rev-erb alpha* [8]–[10] or that closed by the clock-controlled PAR-bZIP proteins through D-Boxes [11]–[13] add to the robustness of the clock mechanism [14].

Patterns of rhythmical transcription depend heavily on post-translational mechanisms operating within the circadian clock [3], [15]–[17]. Post-translational modifications of clock proteins include phosphorylation [16], SUMOylation [18] or acetylation [19], most of which are induced upon formation of heterodimers or larger protein complexes. These processes control intracellular trafficking of clock proteins, their functionality and ultimately their degradation.

A timely orchestrated degradation of clock proteins is indispensable to the proper functioning of the clock as illustrated by the recently uncovered role for the F-Box protein Fbxl3. In mammals, the F-Box protein family comprises about 40 members, which direct ubiquitination and proteasome-mediated degradation of specific proteins through SCF (Spk/Cullin/F-Box protein) E3 ubiquitin ligase complex [20]–[22]. Accordingly, the SCF^Fbxl3^ complex was recently shown to direct degradation of CRY1 and CRY2 proteins, with mutations in Fbxl13 leading to a slowed circadian clock in mice [23]–[25].

We sought to test whether the regulating effect of Fbxl3 was conserved in the diurnal sheep and investigate whether its closest homologue, Fbxl21 [26], is also involved in circadian function.

## Results and Discussion

### Ovine orthologues of Fbxl3 and Fbxl21

**Figure 1:**
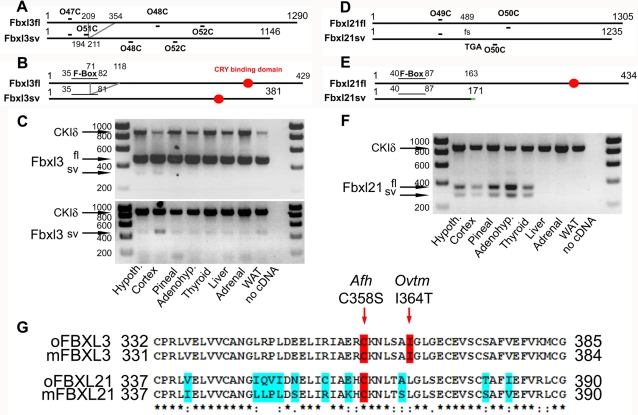
Cloning and mRNA distribution of ovine Fbxl3 and Fbxl21. A/D. Schematics depicting the cds of mRNA of Fbxl3 and Fbxl21, respectively. Primers used for RT-PCR are indicated. Note that Fbxl3sv is an in-frame splice-variant while Fbxl21sv is an out-of-frame splice-variant (fs for frame-shift) with an early stop-codon (TGA, nt 489). B/E. Schematics depicting the proteins encoded by the Fbxl3 and Fbxl21 transcripts, respectively. Note that part of F-Box motif of Fbxl21sv is disrupted due to the splicing event while the CBD is spared. Note also that Fbxl21sv is a short protein product of 171 amino acids with the last 8 amino acids (indicated in green) differing from Fbxl21fl due to the AS event. The F-Box motif is left intact but Fbxl21sv does not have a CBD. C. RT-PCR for Fbxl3 on central and peripheral tissues sampled at ZT4. Co-amplification of a CKIδ fragment was used as an internal positive control (oCKIδ, GenBank EF643522), absence of cDNA in the PCR mix as a negative control. Upper panel: screening with O47C/O48C primers. Lower panel: screening with O51C/O52C primers clarifies the tissue distribution of Fbxl3sv. F. RT-PCR for Fbxl21 on central and peripheral tissues sampled at ZT4. G. Sheep - mouse amino acid conservation within the CBD of Fbxl3 and Fbxl21. Residues associated with the *Afterhours* (*Afh*) and *Overtime* (*Ovtm*) Fbxl3 mutants are highlighted in red. Blue shading indicates residues that differ in at least one of the four sequences. Star, two dots and one dot indicate identical, conservative and semi-conservative substitutions, respectively. Pfam scores for F-box motifs: Fbxl3fl 3.9e^−09^, Fbxl3sv 2.1e^−05^, Fbxl21fl and Fbxl2sv 6.5e^−11^.

Cloning of ovine Fbxl3 generated a full-length (oFbxl3fl, GenBank EF643523) coding sequence (cds) of 1290 nucleotides (nt), giving a protein of 429 amino acids (**Fig 1A/B**). Overall sequence homologies to bovine, murine and human Fbxl3 were all >95%, with 100% amino acid conservation in the region 332–385, which encodes a CRY binding domain (CBD, **Fig 1G**). Additionally, we isolated a truncated in-frame splice-variant (oFbxl3sv, GenBank EF643524), in which nt 209 to 354 are deleted (**Fig 1A**). Alternative splicing (AS) describes the process of splicing exons of pre-mRNAs in different arrangements and drastically expands the potential repertoire of protein variants [27]–[30]. Since there is an evolutionary trend towards increased AS in complex organisms, this mechanism may resolve the paradoxical lack of increasing number of expressed genes in increasingly complex eukaryotic organisms [27]–[28]. It appears that AS is widespread since recent large-scale analyses indicate that as much as 70–80% of genes in mammals are subject to AS [29], [31]. In the case of oFbxl3sv, AS spares the open reading frame but leads to a truncation of the F-box motif in the protein (**Fig 1B**, Pfam score diminished by 2.10^4^-fold compared to oFbxl3fl), while the CBD is left intact. Such a protein is therefore predicted to have intact CRY binding while coupling with the proteasome degradation pathway may be impaired. We designed primers to encompass the predicted AS region (**Fig 1A**, O47C/O48C), or to select for the splice-variant (oFbxl3sv) (**Fig 1A**, O51C/O52C, see M&M for oligos sequences and details), and screened for Fbxl3 expression in ovine tissues (**Fig 1C**). This clearly demonstrates that both oFbxl3fl and oFbxl3sv are ubiquitously expressed.

Sequence phylogeny analysis shows that Fbxl21 is the closest relative of Fbxl3 [26]. We therefore cloned a full-length cds for ovine Fbxl21 (oFbxl21fl, GenBank EU239380). This yielded a 1305 nt sequence giving a protein of 434 amino acids with a greater degree of inter-species divergence than was the case for Fbxl3 (94, 84 and 82% homology with bovine, mouse and human Fbxl21 respectively, **Fig 1D**). Fbxl21 appears to be a paralogue of Fbxl3 that arose through gene duplication relatively recently; indeed, unlike Fbxl3, no clear orthologue of Fbxl21 could be identified in fish genomes (Ensembl database). Within the region corresponding to the CBD of Fbxl3, amino acid identity to mFbxl21 was only 79% although most substitutions were either conservative or semi-consevative (**Fig 1D/E** and **G**). Here too, a splice-variant (oFbxl21sv, GenBank EU239381) was isolated. This splice-variant lacks 70 nt, is therefore out of frame (**Fig 1D/E**; fs: frame-shift) and presents an early stop-codon (TGA). This transcript yields a predicted protein of only 171 amino acids, leaving the F-box motif intact but lacking the CBD (**Fig 1D/E**). Such a protein is therefore unlikely to bind CRY proteins while coupling with the proteasome degradation pathway would be retained. As for oFbxl3, we designed primers (O49C/O50C) to determine the tissue distribution of oFbxl21fl and oFbxl21sv. This revealed that Fbxl21 exhibited much more varied levels of expression between tissues than Fbxl3, with maximal expression in the adenohypophysis, hypothalamus and pineal (all neuroendocrine structures associated with timing and homeostasis) and no expression evident in liver, adrenal or white adipose tissue (WAT) (**Fig 1F**). The truncated transcript, oFbxl21sv, was detected in all tissues in which oFbxl21fl was also expressed. A similar distribution pattern was observed when RNA were extracted from animals sampled at either ZT4 (present data) or ZT15 (data not shown) indicating that absence of detection of Fbxl21 in liver, adrenal and WAT is unlikely due to a time-of-day effect on sampling (see below).

Transcripts encoding truncated protein products such as oFbxl21sv have long been considered as erroneous splicing events but recent evidence indicates they may have specific functions [27], [30], [32]. Strikingly, conservation in splice-variant isoforms between mouse and human is very low, with estimates around 10–20% [28]–[29]. This might indicate meaningful evolutionary differences or, alternatively, reveal that splice-variants are merely a reflection of molecular noise generated by the splicing machinery [28]–[30]. In this context it is noteworthy that the same in-frame Fbxl3sv transcript characterized in the sheep is also present in the mouse (data not shown). Furthermore, we cloned a novel splice-variant for Fbxl21 in the mouse that encodes for a truncated protein product of a sequence reminiscent to that of oFbxl21sv (GenBank EU770600). These data demonstrate that AS is commonplace in the F-box protein family as emphasized elsewhere [26], which offers another putative layer of fine-tuning in the circadian clock. Furthermore, the fact that oFbxl3fl and oFbxl3sv are ubiquitously expressed while oFbxl21fl and oFbxl21sv display a clear-cut tissue-specific distribution strengthens the view that circadian clocks among tissues might have different characteristics, as previously suggested [7], [33].

### Fbxl3 and Fbxl21 physically associate with Cry1

**Figure 2:**
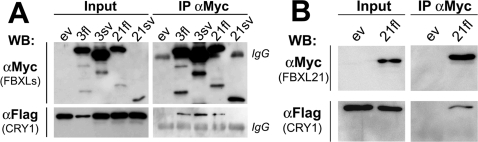
Ovine and murine Fbxl3 and Fbxl21 physically associate with oCry1. A. Extracts from COS7 cells co-transfected with Myc-Fbxl and Flag-Cry1 expression vectors were submitted to immunoprecipitation (IP) with α-Myc beads followed by western-blotting (WB). The oCRY1 protein was readily co-purified with oFbxl3fl, oFbxl3sv and oFbxl21fl but not with oFbxl21sv. Note the low CRY1 levels in the input when Fbxl3fl was present. Similar detection levels of IgG (from Myc antibodies) across all conditions demonstrate loading of comparable amounts of supernatant. ev = empty expression vector. B. The use of similar methods demonstrates that the mouse Fbxl21 also interacts with oCRY1.

We used co-immunoprecipitation assays to determine whether sheep Fbxl3/21 orthologues bind to CRY1 *in-vitro*. We therefore cloned a full-length cds for ovine Cry1 (oCry1, GenBank EF494243) in a Flag-tagged expression vector while Fbxl3fl, Fbxl3sv, Fbxl21fl and Fbxl21sv were cloned in Myc-tagged expression constructs. These were co-transfected into COS7 cells in different combinations, prior to co-immunoprecipitation with beads coated with α-Myc antibodies followed by Western blotting (WB) with α-Myc or α-Flag antibodies (WB, **Fig 2A**). Consistent with data in the mouse [23], [25], we observed pull-down of oCRY1 by oFbxl3fl. This was also observed for oFbxl3sv, as predicted by the preservation of the CBD in this transcript. A similar pull-down was observed for oFbxl21fl, but not for oFbxl21sv that lacks the CBD. These findings confirm predictions and indicate that interaction with CRY1 requires the CBD.

In spite of the sequence divergence between the Fbxl21 CBD of sheep and mouse (**Fig 1G**), pull-down experiments using mFbxl21 instead of oFbxl21 demonstrate that the CRY1 binding ability is intact (**Fig 2B**).

### Differential effects of ovine Fbxl variants on CRY1 protein degradation

**Figure 3:**
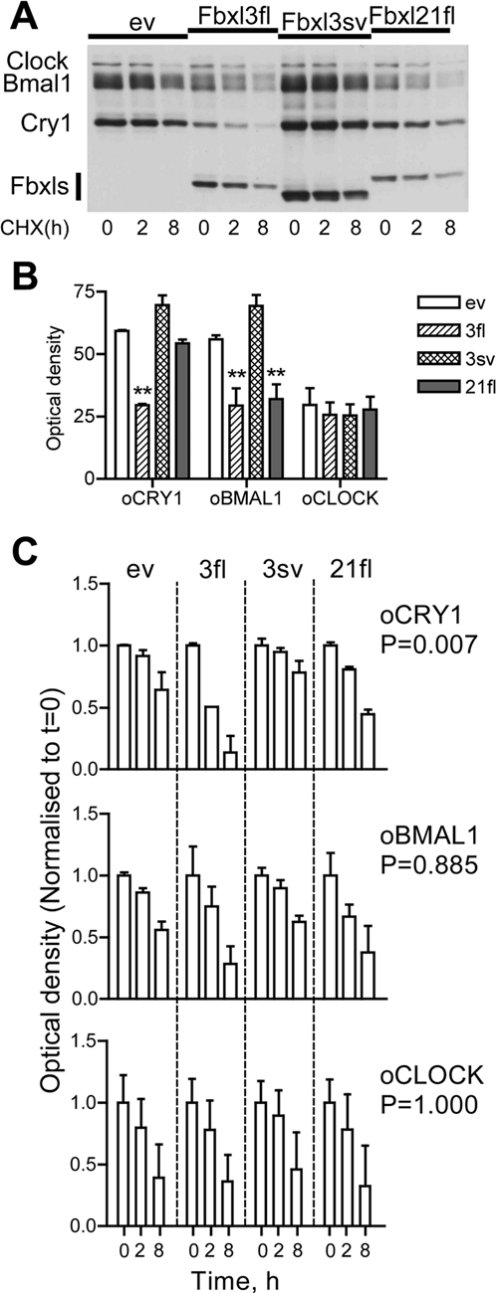
Fbxl21 and Fbxl3 promote degradation of ovine CRY1 and BM1. A. COS7 cells were transfected with oCry1, oBm1 and oClk, and an oFbxl expression vector as indicated above each lane. Extracts were made at the start of cycloheximide (CHX) treatment (t = 0) or 2 or 8 h later as indicated and protein levels assessed by WB. Transfection conditions *per* well: 1 µg of oClock, 600 ng of oBmal1, 600 ng of each oFbxl/ev and 400 ng of oCry1. B. Quantitation of baseline levels (t = 0) of oCRY1, oBM1 or oCLK proteins. Data are normalised to values observed in cells not transfected with any oFbxl (ev). **: significantly lower density than for ev, p<0.01, One-way ANOVA followed by Student-Newman-Keuls post-hoc test. C. Quantitation of temporal decline in protein levels following the start of CHX treatment. Data are expressed relative to t = 0 values for each vector combination. p values for the interaction (treatment×time, two-way ANOVA) are given on the right. Data in B/C are mean±SEM of observations from 3 replicate experiments.

We then assessed whether oFbxl proteins affect the degradation kinetics of molecular clock components. For this purpose full-length cds for orthologues of Clk (oClk, GenBank EU016223) and Bm1 (oBm1, GenBank EF494245) were cloned in the sheep. COS7 cells were co-transfected with oCry1, oBm1 and oClk in combination with the three Fbxl constructs which co-immunoprecipitated with oCRY1, and then treated with the protein synthesis inhibitor cycloheximide (CHX, 25 µg/ml) for 0–8 h. Proteins were extracted and relative levels were assessed by WB (**Fig 3A**).

The Fbxl proteins had different effects on baseline oCRY1 and oBM1 levels immediately prior to CHX treatment (t = 0), but no impact on those for oCLK (**Fig 3B**). Full-length oFbxl3 strongly reduced baseline of oCRY1 levels while oFbxl21fl did not have any clear effect on oCRY1 levels, in agreement with previous results (see **Fig 2A**, input). Contrastingly, baseline levels of oCRY1 showed a trend towards higher levels by co-transfection of oFbxl3sv, in-line with the predicted impaired coupling to the proteasome degradation pathway and suggestive of a dominant-negative effect.

To normalise for these differing baseline levels, data were replotted as a fraction of t = 0 values for each expression vector combination (**Fig 3C**). Degradation rates in cells co-transfected with empty vector (ev) served as controls. These data demonstrate that only the rate of oCRY1 degradation is highly dependent on co-transfection of oFbxl expression vectors (two-way ANOVA, the value for the interaction treatment×time is indicated on the right of **Fig 3C**). When compared to the ev control condition it was clear that oFbxl3fl exerts the strongest degradation promoting effect on CRY1 proteins (post-hoc test, p<0.01), reducing their levels to less than 25% of t = 0 values after 8-h of protein synthesis blockade. A qualitatively similar but quantitatively more modest effect (non-significant, post-hoc test p<0.1) was seen for oFbxl21fl. By contrast, degradation rates for oCRY1 (but not oCLK or oBM1) in cells transfected with oFbxl3sv showed a trend towards reduction compared to controls (non-significant, post-hoc test p<0.1), confirming predictions that the truncated F-box motif in this protein leads to a protein stabilising effect.

Collectively these data indicate that Fbxl21fl and Fbxl3sv, in addition to Fbxl3fl, govern the kinetics of degradation of CRY1.

### Fbxl3 and Fbxl21 modulate CRY1 suppression of CLK/BM1 induced E-box transactivation

To investigate whether these differential effects on protein stability result in different clock gene function *in vitro*, we constructed a luciferase reporter with the promoter region of the sheep *Rev-erb α* gene (GenBank EF494248), a gene whose transcription is regulated through circadian E-boxes, responsive to CLK/BM1 [1]–[3]. We then performed further co-transfection experiments in which the impact of the various Fbxl on oCRY1-dependent transcriptional repression of *oRev-erb α* expression could be assessed.

**Figure 4:**
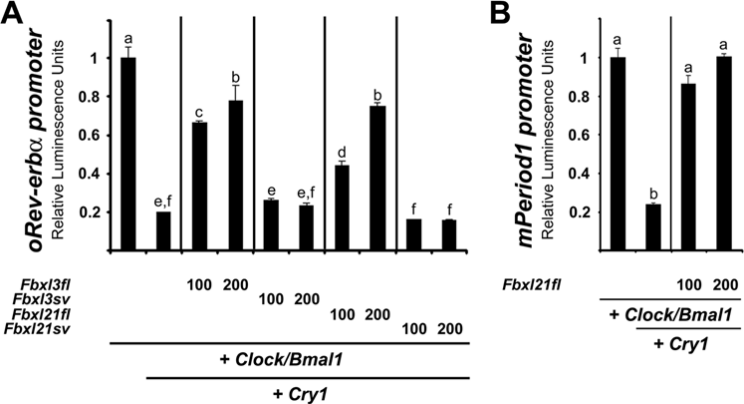
Functional transcriptional effects of Fbxl3 and Fbxl21. A. oFbxl3 and oFbxl21 dose-dependently reverse oCRY1 inhibition of oCLK/oBM1 induced transactivation of an *oRev-erb α* reporter construct. Transfection conditions *per* well: 100 ng of oClock, 100 ng of oBmal1, 100 ng of oCry1, 100 or 200 ng of each Fbxl or ev, 50 ng of *oRev-erb alpha* promoter reporter, 50 ng of b-Gal reporter and/or necessary amount of empty vector to make a final amount of 600 ng. B. mFbxl21 dose-dependently reverses mCRY1 inhibition of mCLK/mBM1 induced transactivation of an *mPer1* reporter construct. Transfection conditions *per* well: 100 ng of mClock, 100 ng of mBmal1, 20 ng of oCry1, 100 or 200 ng of mFbxl21 or ev, 50 ng of *Per1* promoter reporter, 50 ng of b-Gal reporter and/or necessary amount of empty vector to make a final amount of 600 ng. Data in A/B are mean±SEM of one triplicate representative from 3 replicate experiments. Different letters above bars indicate significantly different groups (p<0.05).

Similarly to results obtained in the mouse [25], this approach revealed that oCRY1 is a potent inhibitor of oCLK/oBM1-induced *oRev-erb α* transactivation, reducing expression by approximately 5-fold (**Fig 4A**). This repressive effect was attenuated by approximately 75% upon co-transfection with oFbxl3fl or oFbxl21fl, consistent with the oCRY1 degradation promoting effects of these proteins. According to predictions, oFbxl3sv and oFbxl21sv proteins failed to reduce the repressive effects of oCRY1, likely due to the truncated F-box motif and lack of the CBD, respectively.

A similar set of experiments was performed using previously validated [34] murine clock components (mClk, mBm1, mCry1 and an *mPer1-luc* reporter gene construct). Akin to what is observed for oFbxl21fl, mFbxl21fl is able to reverse mCRY1-induced repression of mCLK/mBM1 transactivation (**Fig 4B**).

### Rhythmical Fbxl21 expression is confined to the SCN within the hypothalamus

**Figure 5:**
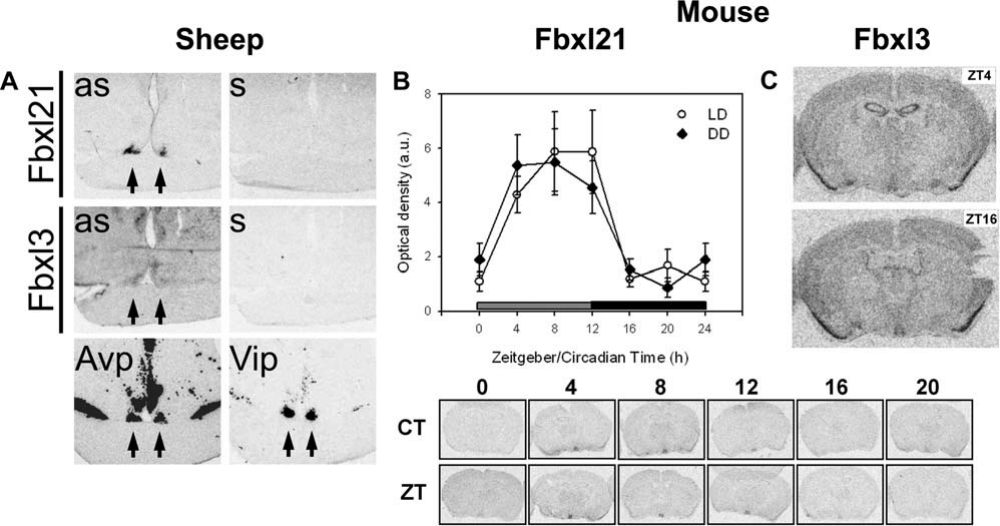
*In-situ* hybridisation for Fbxl3 and Fbxl21 within the SCN of the sheep and mouse. A. Representative images for expression of Fbxl21 (top), Fbxl3 and the neuropeptides, Avp and Vip (bottom) in the hypothalamus of sheep sampled at ZT4-6. Sense controls for Fbxl riboprobes are also shown (top and middle right panels). B. Fbxl21 undergoes circadian expression in the SCN of the mouse. Top: mRNA profiles in the SCN of mice sampled at 6 different time-points (n = 5 per time-point) under either a 12/12 light-dark cycle (LD) or under constant darkness (DD). Data for ZT/CT0 are double-plotted. Bottom: representative *in-situ* autoradiograms. Note the SCN-restricted pattern of expression. Two-way ANOVA: time-effect, P<0.001, light condition×time interaction, p = 0.39. C. Fbxl3 does not show circadian expression in the SCN of the mouse. Representative images for expression of Fbxl3 in the brain of mouse sampled at ZT4 and ZT16. Note the ubiquitous pattern of labelling including diffuse expression in the SCN.

Our RT-PCR screen showed that Fbxl3 is ubiquitously expressed while Fbxl21 has a highly tissue-specific expression pattern. We therefore wondered if Fbxl21 also shows localised expression within the hypothalamus. To address this, we performed radioactive *in situ* hybridisation in brain tissue from sheep sampled at ZT 4-6 and mice sampled around the clock using homologous probes. Remarkably, in the sheep hypothalamus we observed strong labelling within the SCN, which harbours the mammalian master clock, in a region overlapping with sites of arginine vasopressin (Avp, GenBank EU045357) and vaso-active intestinal polypeptide (Vip, GenBank EU016225) expression (**Fig 5A**). This pattern contrasts markedly with that for Fbxl3, which showed relatively weak and diffuse labelling (**Fig 5A**). Further experiments in mice confirmed this SCN-restricted pattern of Fbxl21 expression, and demonstrated pronounced diurnal and circadian expression rhythms, rising rapidly at the start of the day (or “subjective day” in animals run into constant darkness), and declining at the onset of the (subjective) night (**Fig 5B**). In contrast, Fbxl3 was diffusely expressed in the SCN of mice where it displayed no diurnal or circadian oscillation (data not shown and **Fig 5C**), in-line with previous findings [24], [25]. Representative hybridization images from brains of animals sampled either in the beginning/middle of the day (ZT4) or beginning/middle of the night (ZT16) are shown in **Fig 5C**.

These data suggest that Fbxl21 may be a clock-controlled gene that plays a specific role in SCN pacemaker function.

### Fbxl21 is a clock-controlled gene

**Figure 6:**
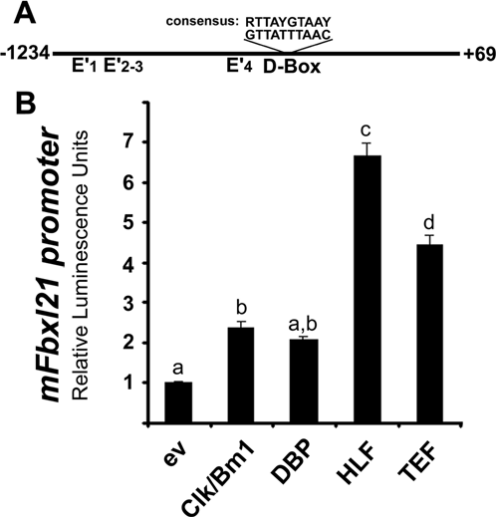
mFbxl21 is a clock-controlled gene. A. Schematics depicting the promoter region of the mouse *Fbxl21* gene, E' indicate non-canonical E-Box motifs, D-Box indicates a putative response element for the PAR-bZIP proteins. B. The mouse *Fbxl21* gene is responsive to both CLK/BM1 and the PAR-bZIP transcription factors. Transfection conditions per well: 50 ng of *Fbxl21* promoter reporter, 50 ng of b-Gal reporter and either 100 ng of mClock+100 ng of mBmal1 or 100 ng of each of the PAR-bZIP transcription factors and/or necessary amount of empty vector to make a final amount of 300 ng. Data in A/B are mean±SEM of one triplicate representative from 3 replicate experiments. Different letters above bars indicate significantly different groups (p<0.05).

The circadian pattern of Fbxl21 expression in the mouse SCN is reminiscent of that seen for other genes whose expression is driven by CLK/BM1 such as *Per1* or *Dbp*. Analysis of the *mFbxl21* gene promoter revealed no canonical E-Box (CACGTG) but 11 E' Boxes (CANNTG). Only 4 of these are conserved in the rat promoter (**Fig 6A**, E'1–4). Interestingly we noticed a putative D-Box element (**Fig 6A**), which is also conserved in the rat, human, bovine, dog and chicken *Fbxl21* gene promoters in spite of the very poor sequence conservation between species (data not shown). Such a response element is known to mediate transactivation by members of the PAR-bZIP transcription factors family (DBP: albumin D-site binding protein, GenBank EU293835; HLF: Hepatic leukemia factor, Genbank EU293836 and TEF: thyrotroph embryonic factor, GenBank EU293837) that are clock-controlled genes and display strong circadian oscillation within the SCN peaking early into the light phase [12]–[13]. We therefore cloned a 1.3 kb fragment of the proximal *mFbxl21* gene promoter and used luciferase assays to assess transactivation by CLK/BM1 and the PAR-bZIP proteins (**Fig 6B**). This revealed modest (∼2-fold) transactivating effects of CLK/BM1 and DBP, and stronger (4–7 fold) effects of HLF and TEF. The modest (n.s., p = 0.08) effect of DBP could be due to differences in binding affinities of the various PAR-bZIP proteins towards this D-Box [35]. Alternatively, transcriptional co-factors required for DBP might be missing in this cell type.

Taken together, these data suggest that the rhythmic expression of Fbxl21 in the mouse SCN derives from combined transactivation through E'boxes and a conserved D-Box element.

### Conclusion

**Figure 7:**
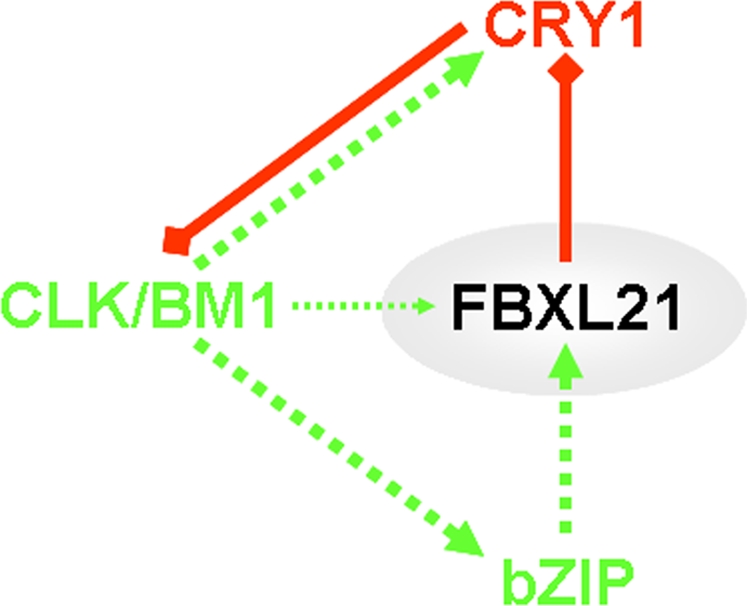
A model for Fbxl21 function in the mammalian circadian clock. Transcriptional control (green dashed line) by CLK/BM1 directs rhythmic expression of *Cry1* and the PAR-bZIP and also has a modest effect upon *Fbxl21*. Transcription of *Fbxl21* mostly depends on the PAR-bZIP proteins. The FBXL21 protein in turn controls degradation of CRY1 (red diamond-ended line) and therefore exerts a pivotal role in the control of CRY1-mediated inhibition of CLK/BM1 transcription (red diamond-ended line).

Altogether, these data indicate a broad conservation of transcriptional and post-translational mechanisms of the circadian clock between a nocturnal rodent and a diurnal ungulate and identify Fbxl21 as a new clock component whose expression is driven by the core-clock. The functional characterization of new splice-variants also suggests that AS might be an important mechanism to fine-tune the clock. This idea is also supported by data for Bmal2 [34], [36], Timeless [37] or the finding of a temperature-dependent splicing of the *Period* gene in *Drosophila* [38]. Since Fbxl21 directs degradation of CRY1 and displays strong and specific rhythmic circadian expression within the central pacemaker of the SCN, we suggest that Fbxl21 stands at a crucial crossroads between activation and repression in central clocking (**Fig 7**). Finally, the present data also raise the possibility of functional redundancy between Fbxl3 and Fbxl21 in the SCN akin to what was recently reported for Clock and Npas2 [39]. Future studies defining FBXL21 protein profiles within the SCN and targeted deletion of the gene should help delineate further the role of Fbxl21 in circadian timing.

## Materials and Methods

### Sheep tissue collection

Hypothalamic blocks for *in-situ* hybridisation and central tissue samples were obtained from Blackface sheep killed at a local slaughterhouse (McIntosh Donald, Portlethen, UK). Sheep were killed by the pneumatic captive bolt gun method carried out by a licensed person. Peripheral tissues were sampled from Soay rams killed by an overdose of barbiturate (Euthatal; Rhone Merieux, Essex, UK) administered intravenously. All experiments were performed in accordance with Home Office (UK) legislation and approved by University of Edinburgh Local Ethics Committee. Tissues were rapidly dissected out, frozen onto dry ice and kept at −80°C until sectioning or RNA extraction.

### Mouse tissue collection

Adult male Swiss mice (Charles River, Lyon, France) were kept in a 12-h light/dark cycle (LD) or transferred to constant darkness (DD) for 2 days. After mice were deeply anesthetized with isoflurane and decapitated, brains were removed, frozen and cut in 20-µm coronal sections at the level of the suprachiasmatic region. Five mice were killed every 4 h throughout the LD cycle (expressed in zeitgeber time, ZT0 being the time of lights on) or throughout the DD cycle (expressed in circadian time, CT0 being defined as the projected time of lights on). Experiments were performed in accordance with the rules of the European Committee Council directive of November 24, 1986 (86/609/EEC) and the French Department of Agriculture (license n°67–88 to E.C.).

### Cloning

DNA extraction was done with a QIAamp DNA mini kit (Qiagen). RNA extraction was performed using Tri-reagent (Sigma) according to the manufacturer's protocol, and cDNA synthesis was carried out using a reverse transcription kit (Qiagen). When sheep EST were not available, ClustalW alignments of mammalian sequences were performed in order to identify conserved regions suitable for primer design (primers from MWG Biotech, Germany). PCR was done with Platinum Taq Hifi (Invitrogen) according to the manufacturer's protocol. Following agarose gel electrophoresis, PCR fragments of the expected sizes were extracted using a gel extraction kit (Qiagen) and cloned in pGEM-T easy vector (Promega). Four to six positive clones were sequenced (MWG, United Kingdom), and the sequences deposited in GenBank. To generate expression constructs, a second round of PCR was performed using primers flanked by adequate restriction sites and the pGEM-T clone as template. PCR fragments were extracted as described above, digested by the adequate restriction enzymes, purified with a PCR purification kit (Qiagen) and cloned in the expression vector backbone. Two different expression vectors with either a Flag or a 5X-Myc tag were used. To generate promoter reporter constructs, a strategy identical to that described above was applied. All fragments were cloned into the pGL3 basic backbone (Promega) digested with the appropriate restriction enzymes. Sequencing was performed to check accuracy of the re-amplified cloned fragments. All primer sequences and constructs are available upon request.

### Cell culture, transfection and luciferase assay

COS-7 cells were grown in Dulbecco's modified Eagle's medium supplemented with 10% fetal bovine serum, 1% Pen/Strep mix and sodium pyruvate in a humidified atmosphere with 5% CO2 at 37°C. Cells were plated in either 6-well or 24-well plates (for WB or luciferase assay, respectively), and transfected with GeneJuice (Novagen). For luciferase assays, expression vectors were used at different doses depending on the experiment. Promoter reporter constructs and *β*-galactosidase reporter construct were used at 50 ng/well. Total transfected DNA amount was set to an equal amount between all conditions by addition of the corresponding empty vector. Luciferase assay was performed on the day following transfection. Briefly, cells were rinsed twice in cold PBS and lysed for 15 min in lysis buffer (25 mM Tris, 2 mM EDTA, 1 mM DTT (dithiothreitol), 10% glycerol and 1% Triton X-100). The luciferase assay was performed using a Luciferase assay system kit (Promega) and a Lumicount microplate luminometer (Packard). Results were normalised to *β*-galactosidase activity. Data (in relative luminescence units) represent fold induction once normalised to *β*-galactosidase. Experiments were repeated three to five times, each condition in triplicate. For WB experiments, variable amounts of DNA were transfected, never exceeding 4 µg/well. Experiments were done at least in triplicate.

### Western blot and immunoprecipitation

Cells were plated in 6-well plates and transfected as described above. For protein synthesis inhibition experiments, 25 µg/ml cycloheximide (CHX; Sigma) was added 24 h post-transfection for 8, 2 or 0 h prior to harvesting. Cells were rinsed twice in cold PBS, resuspended in RIPA buffer (50 mMTris-HCl, pH 8, 150 mM NaCl, 1% Nonidet P40, 0.5% Na-deoxycholate, 0.1% SDS, 0.5 mM DTT, 1 mM PMSF and a cocktail of protease inhibitors (Complete Mini; Roche) and incubated for 30 min on ice. After vortexing, the extract was centrifuged for 1 min at 16000 g and supernatants used for Western blot (WB) or kept at −80°C. For immunoprecipitation assay cells were harvested 48 h post-transfection in IP buffer (150 mM NaCl, 5 mM EDTA, 0.5% Nonidet P40, 50 mM Tris, pH 7.8, 1 mM PMSF and a cocktail of protease inhibitors (Complete Mini; Roche). An aliquot of supernatant was kept as input for western-blot analysis while the rest was incubated overnight at 4°C with anti-Myc affinity resin (Sigma) in IP buffer. The beads were then washed twice with IP buffer, resuspended in Laemmli buffer (50 mM Tris/HCl pH 6.8, 2% SDS, 0.1% Bromophenol Blue, 10% glycerol and 100 mM DTT), denatured at 95°C for 5 min and the supernatant was resolved on a 6% or 12% SDS polyacrylamide gel. Nitrocellulose membranes (Amersham) were then probed with antibodies directed against Myc (Roche) or Flag (Sigma). Anti-mouse horseradish peroxidase-conjugated antibody (Sigma) and the ECL® (enhanced chemiluminescence) detection reagent (Amersham) were used for revelation using Hyperfilm ECL (Amersham).

### RT-PCR on tissues

RT-PCR was performed as described in the tissue collection and cloning section. Co-amplification of an 879 bp fragment of oCKIδ was used as a positive control. Omission of cDNA in the PCR mix was used as a negative control. Initial screening for Fbxl3 was performed using oligos O47C (GGGGTAATCTCCTTCAGG) and O48C (CTAGGACTTTGAGCGAGG) yielding 471 and 327 nt PCR fragments for Fbxl3fl and Fbxl3sv, respectively. Another PCR was performed using oligos O51C (GCAACTGGAACCAGGTGG ) and O52C (GGAAGTGAGTCTGTCCTGG) yielding a 474 nt PCR product for Fbxl3sv only since O51C spans the splicing junction (the sequence corresponding to O51C in Fbxl3fl being GCAACTGGAACCAGGTAT prevents complete hybidization of the oligo hence polymerisation). Confirming specificity of the approach, no PCR product was obtained with O51C/O52C when the Fbxl3fl expression vector was used as a template while the expected 474 nt product was readily amplified from the Fbxl3sv expression construct. PCR screening for Fbxl21 was performed using oligos O49C (CCATCTCCAGTATGTCAGC) and O50C (ATGCCAGTTCTCTCAGACC) yielding 386 and 298 nt PCR fragments for Fbxl21fl and Fbxl21sv, respectively.

### *In-situ* hybridisation

Complete cds for the ovine arginine-vasopressin (Avp) and vasoactive intestinal polypeptide (Vip) were cloned and deposited in GenBank. Homologous probes used in this paper are as follows: oFbxl3 covers nt 110–580 of EF643523, oFbxl21 covers nt 342–709 of EU239380, oAvp covers nt 1–501 of EU045357, oVip covers nt 1–513 of EU016225, mFbxl3 covers nt 450–1175 of NM_015822, mFbxl21 covers nt 905–1656 of NM_178674. Hypothalamic blocks for *in situ* hybridisation were cut into 20 µm sections using a cryostat, and thaw-mounted onto poly-lysine and gelatin coated slides. Radioactive cRNA riboprobes were prepared by plasmid linearisation and *in vitro* transcription reactions including ^35^S-UTP (Perkin-Elmer). Sections were hybridized overnight at 60°C with 5.10^5^ cpm of probe *per* slide, subjected to RNaseA digestion and stringency washes in sodium citrate buffer to remove nonspecific probe hybridisation. Slides were then dehydrated in graded ethanol solutions and exposed to an autoradiographic film (Kodak) for 4 days.

### Statistics

Data were analysed (Sigma-stat, Jandel Scientific) by one-way or two-way ANOVA followed, when appropriate, by the Student-Newman–Keuls post-hoc test. Significance was set at P<0.05.
